# The influence of big data analytic capabilities building and education on business model innovation

**DOI:** 10.3389/fpsyg.2022.999944

**Published:** 2022-10-18

**Authors:** Yong Cui, Saba Fazal Firdousi, Ayesha Afzal, Minahil Awais, Zubair Akram

**Affiliations:** ^1^Overseas Education College, Jiangsu University, Zhenjiang, China; ^2^School of Management, Lahore, School of Economics, Lahore, Pakistan; ^3^School of Management, University of Agriculture Faisalabad, Faisalabad, Pakistan

**Keywords:** business model innovation, technological orientation, employee creativity, big data analytical capabilities building and education, China

## Abstract

As organizations are benefiting from investments in big data analytics capabilities building and education, our study has analyzed the impact of big data analytics capabilities building and education on business model innovation. It has also assessed technological orientation and employee creativity as mediating and moderating variables. Questionnaire data from 499 managers at enterprises in Jiangsu, China have been analyzed using Structural Equation Modeling (SEM) in SmartPLS. Big data analytics capabilities building and education strengthen technological orientation and increase business model innovation. Technology orientation increases business model innovation and plays a mediating role. Employee creativity also boosts innovation. These findings show that business managers should adopt and promote a technological orientation. They should hire and train employees with big data education and training. Organizations can try to select and support employees who show creativity.

## Introduction

Big data has revolutionized old business models (McAfee et al., 2012). The main premise big data analytics builds on is that by analyzing large amounts of data from multiple sources, actionable insights can be extracted that can help businesses achieve an edge over rivals (Chen et al., 2012; Behl et al., 2022; Edu, 2022). Big data can provide valuable information (Hofacker et al., 2016) and provide competitive advantages (Morabito, 2015). Research has found that enterprises that use big data analytics can achieve higher levels of performance, in terms of innovation (Khan and Tao, 2022; Zheng et al., 2022). This is why organizations are making large-scale investments in big data analytics (SAS, 2013; Sharma et al., 2014). To find out more about this topic, big data analytics capabilities have been included in our research model as an antecedent of business model innovation.

Innovation is consistently ranked amongst the top priorities of business executives (Teece and Leih, 2016). The possibility to utilize big data analytics capabilities to pursue innovative strategies (Ciampi et al., 2020; Munir et al., 2022) is generating radical changes in business logics in many industries (Wang and Hajli, 2017; Santoro et al., 2018). To obtain more knowledge, business model innovation has been included as an outcome of big data analytics capabilities in our model.

A growing number of enterprises are making efforts to attain durable competitive advantages by utilizing technologies for business model innovation (Spieth et al., 2019). With support from technology, big data has emerged as the main tool helping businesses in exploitative and explorative activities (McAfee et al., 2012). As technology supports innovation (Dong and Yang, 2019), business capabilities can be utilized more productively if an organization has a technological orientation. Therefore, we have included technology orientation as a mediating variable in the relationship between big data analytics capabilities and business model innovation.

In this context, employee behavior that contributes to production and application of new ideas is favorable because it leads to organizational innovation (Van de Ven, 1986; Amabile, 1988; Scott and Bruce, 1994; Janssen, 2000; Shanker et al., 2017). This shows that dynamic capabilities, including those related to big data, are likely to generate more benefits if an organization has a creative workforce. So this research model includes direct and moderating effects of employee creativity.

The ongoing COVID-19 pandemic is a global challenge (Clark et al., 2020) and businesses need useful recovery mechanisms (Breier et al., 2021) because this crisis will negatively affect existing business models (Ritter and Pedersen, 2020). If major changes are made in the elements of a business model to achieve innovation (Foss and Saebi, 2017), new opportunities can be seized to recover from this crisis (Breier et al., 2021). This means it is important to conduct research to understand the factors affecting business model innovation.

For this research, we have selected China as the appropriate context. World Development Indicators show that China’s research and development expenditure increased 21.90% from 2011 to 2020. The country’s innovation index increased 14.87% over the same period. This shows that creativity and business model innovation are important areas for organizations in China so should be investigated.

The key objectives of our research are to analyze effects of big data analytics capabilities on business model innovation and technological orientation. They include assessing the mediating role of technological orientation and moderating effect of employee creativity. This framework is supported by the dynamic capabilities view. For our study, an online questionnaire was administered to mid-level and senior managers working at Chinese organizations. Data were analyzed using Structural Equation Modeling (SEM). It was found that big data analytics capabilities strengthen technological orientation and increase business model innovation. Technology orientation increases innovation and plays a mediating role in the relationship between big data capabilities and innovation. Employee creativity boosts business innovation. Creativity has a moderating effect on the relationship between big data capabilities and innovation.

This study has made multiple conceptual contributions. It has added to existing literature on the advantages of dynamic capabilities for businesses (Akter et al., 2016; Mikalef et al., 2019). We have responded to calls for more studies on the internal drivers of business model innovation (Martins et al., 2015; Saebi and Foss, 2015; Foss and Saebi, 2017; Frankenberger and Sauer, 2019). These drivers include technological orientation and employee creativity (Ciampi et al., 2021) Further, to the best of our knowledge, no past study has included both mediating and moderating variables in the relationship between big data analytics capabilities and business model innovation. These findings have also provided support for the dynamic capabilities view. To the best of our knowledge, past studies have not analyzed this conceptual model in the Chinese context so this research has made a contextual contribution as well.

This study has also generated valuable managerial implications. Findings show that to achieve business model innovation, managers should adopt and promote a technological orientation. They should also hire and train employees who are most capable of utilizing big data. Organizations should try to select and support employees who show creativity (Ciampi et al., 2021). Further, such workers should be placed in management positions. In this setting, top managers can play an important role (Barton and Court, 2012).

The next section of this paper presents the theoretical background and hypotheses. After that, the research method is described. The next section highlights results obtained. After that, a discussion of the results is provided. Theoretical and managerial implications are presented. Directions for future research are also offered. The paper ends with a conclusion.

## Literature review, theoretical basis, and hypothesis development

### Dynamic capabilities view

Dynamic capabilities are defined as an organization’s abilities for creating, combining and reconfiguring resources to deal with environments that change very quickly (Teece et al., 1997). They include skills and knowledge useful for changing existing resources and generating new value (Teece, 2007; Day, 2014). Dynamic capabilities equip businesses for developing distinguishing procedures and skills, which are essential for retaining success as well as generating new opportunities (Fornell and Larcker, 1981).

The dynamic capabilities view is an appropriate framework for analyzing whether big data analytics capabilities can be leveraged to achieve business model innovation (Ciampi et al., 2021). This view is also appropriate for identifying relevant factors (Ciampi et al., 2021) such as technological orientation and employee creativity. Multiple relevant studies have applied dynamic capabilities as their main theoretical perspective (Akter et al., 2016; Wamba et al., 2017).

In today’s dynamic environment, businesses need to have the capacity to explore as well as exploit changes (Zheng et al., 2011). The dynamic capabilities view highlights the organizational ability to change quickly so it is a suitable theory, in this setting. Successful enterprises need to be data-driven (McAfee et al., 2012) and utilizing big data requires dynamic capabilities (McAfee et al., 2012). Further, dynamic capabilities stimulate innovation (Liao et al., 2009; Tellis et al., 2009; Wei and Lau, 2010). Supporting research has showed that there is a positive relationship between technology usage and organizational performance (Barba-Sanchez et al., 2018). These studies show relevance of the dynamic capabilities view in the contemporary business world. Applying the dynamic capability theory, Li et al. (2022) conducted a study in China. They found that big data analytics positively impact performance. This research highlights relevance of the dynamic capabilities view in the context of China.

The resource-based view is considered one of the most important theories for understanding how organizations can reach higher performance levels (Chae et al., 2014; Hitt et al., 2016a). In the context of technology-oriented businesses also, the theory is useful for improving performance (Cole et al., 2019). This theory states that a business is an amalgamation of different resources and heterogeneity amongst these explains differences in performance (Li et al., 2022). These resources include technological tools such as big data, technology orientation and employee creativity (Li et al., 2022). Keeping these factors in mind, we have selected the dynamic capabilities and resource-based views to support our model.

### The impact of big data analytics capabilities on business model innovation

Big data analytics capabilities are defined as an enterprise’s distinguishing and unique abilities for using big data to obtain strategic insights (Mikalef et al., 2017). Business model innovation is the process of reconfiguring elements in the business value logic (Bucherer et al., 2012). This process requires significant adjustment of at least one core value element so it generates new ways of creating, proposing and/or capturing value (Amit and Zott, 2012). A business model is viewed as successful if it can stay relevant for stakeholders, over time (Gambardella and McGahan, 2010). In this area, businesses can utilize big data analytics to accurately predict environmental changes and update their structures accordingly (Gupta et al., 2020). Analytical data and tools can be utilized to generate business model innovation (Ransbotham and Kiron, 2017). Innovating businesses without the requisite capacities may die even though they are the best at innovation (Teece, 1986).

Supporting research has found that technologies are driving significant innovation in the business world (Mostaghel et al., 2022). Importantly, big data analytics capabilities have a positive impact on business model innovation (Ciampi et al., 2021). Further, the spreading of big data analytics has offered businesses options to upgrade their models (Porter and Heppelmann, 2015). Ransbotham and Kiron (2017) have stated that big data analytics help in detection of new business opportunities by blending diverse sources of data. In this area, big data analytics capabilities improve an organization’s performance in exploration activities (Rialti et al., 2019). They also improve an organization’s speed in generating required responses (Teece et al., 2016) because information flowing freely can clarify what action needs to be taken (Tarafdar and Qrunfleh, 2017). So big data analytics capabilities improve an organization’s capacity for innovation (Kiron et al., 2012, 2014). These dynamic capabilities have been found to positively impact exploratory activities and value creation in Chinese enterprises as well (Shamim et al., 2020).

Importantly, Mikalef et al. (2019) have discovered that big data analytics capabilities have a positive impact on incremental as well as radical innovations. Using big data to understand customers can generate incremental innovation (Story et al., 2011). Big data analytics capabilities can support enterprises in radical innovation, through development of new products or services which can create new markets or radically change existing markets (Erevelles et al., 2016).

In environments that are highly unpredictable, business model innovation can provide opportunities (Giesen et al., 2010) in terms of new methods of generating and capturing value (Amit and Zott, 2010). This is why business model innovation can be a strong response for the COVID-19 crisis. In China, both government and non-government organizations have actively utilized big data technology to prevent and control COVID-19 (Wu et al., 2020). In the country, this technology has played an important role in controlling the disease through tracking, early warnings, medical treatment, resource allocation and recovery efforts (Wu et al., 2020).

Another important contribution of big data analytics capabilities is in the area of environmental welfare. Green management of supply chains has gained a significant amount of attention in academia and business, as environmental awareness has increased (Wang et al., 2020). In China’s manufacturing sector, corporate social responsibility positively impacts green supply chain management and this relationship is strengthened by big data analytics capabilities (Wang et al., 2020). So these capabilities can improve an organization’s capacity to pursue novel and eco-friendly management practices.

Based on this discussion, our first hypothesis is.

*H1*: Big data analytics capabilities have a direct and positive effect on business model innovation

### The impact of big data analytics capabilities on technological orientation

Past research has highlighted the impact of big data analytics capabilities on several strategic orientations (Ciampi et al., 2021). This shows the importance of investigating the association between big data analytics capabilities and a technological orientation. A technological orientation describes an enterprise’s idea openness and inclination to utilize new technologies as well as integrate these into products (Gatignon and Xuereb, 1997; Hurley and Hult, 1998; Li, 2005; Chen et al., 2014; Tsou et al., 2014). In this topic, Mikalef et al. (2018) have found that enterprises possessing big data analytics capabilities can obtain and analyze data for generating insights by utilizing their technology. So these organizations are more likely to have an inclination for technology.

Research has found that a combination of multiple factors, such as technology, is important for value generation through data. This means that big data can lead to a technology orientation. For example, Limbu et al. (2014) have discovered that a technological orientation can play an important role in the relationship between technological capabilities and organizational outcomes. Through technology, traditional business models can be improved and innovation can be achieved (Wang et al., 2021). In harmony with this, Ryu and Lee (2018) have found that big data analytics are used by businesses with a strong technology orientation to support their operations. Similarly, Mandal (2018) has found that big data analytics capabilities can improve performance and this relationship is supported by technology orientation. China’s Social Credit System is designed to centralize and build surveillance infrastructure, using big data from multiple sources (Liang et al., 2018). The goal is to predict and manage the reliability of citizens, organizations and the government (Liang et al., 2018). In this area, the country’s government is improving its surveillance capacity by using technologies (Creemers, 2017; Hoffman, 2018). So big data has led to the generation of a technological orientation.

Based on these findings, our next hypothesis is

*H2*: Big data analytics capabilities have a direct and positive effect on technological orientation

### The impact of technological orientation on business model innovation

Wirtz et al. (2010) have stated that business model innovation is often the outcome of factors such as technology. Multiple studies have found that technology has positive effects on innovation (Morikawa, 2004; Joshi et al., 2010; Kleis et al., 2012; Mithas et al., 2012; Ravichandran et al., 2017; Saldanha et al., 2017; Dong and Yang, 2019). Further, digital transformation provides a boost for business innovation (Wang et al., 2022). In China, technology utilization has a positive impact on business innovation (Chen et al., 2015). So in businesses, a technological orientation is considered important for innovation and maintaining the edge in the market (Dinesh and Sushil., 2019).

Enterprises achieve this innovation through collaboration (Chesbrough and Bogers, 2014). In this area, success depends mostly on trust (Chesbrough and Bogers, 2014). Digital trust, a combination of trust and technology, is a powerful resource for keeping these networks intact (Mubarak and Petraite, 2020). It has been observed that blending knowledge from external sources is important for organizational innovation (Trantopoulos et al., 2017) and technological systems provide help in these operations (Haug et al., 2020). Even when businesses are separated by distance, technology helps them achieve innovation by enabling them to share knowledge (Rai et al., 2006; Nambisan et al., 2017). So technological orientation can be expected to generate business innovation.

Enterprises with this type of orientation are in a better position to develop innovative offerings (Fink and Neumann, 2009). Past studies have revealed that technology can lead to creation of new value propositions, based on better knowledge about customers (Ansari and Mela, 2003; Mithas et al., 2006). However, Hempell and Zwick (2008) have found that technology negatively affects an organization’s ability to innovate. Similar research has revealed that users sometimes respond to technological complexity by working outside these systems (Boudreau and Robey, 2005). This means that knowledge is not shared and innovation processes are negatively affected (Haug et al., 2020). So research has underlined both positive and negative effects of technological orientation, for business model innovation.

Based on this discussion, our third hypothesis is

*H3*: Technological orientation has a direct and positive effect on business model innovation

### The mediating role of technological orientation

Limbu et al. (2014) have stated that an organization’s big data capabilities cannot guarantee improved performance because technological orientation may also be required. Supporting this assertion, research has found that technology systems alone are not sufficient for increasing innovation because technological skills, processes and infrastructure are also needed (Turulja and Bajgorić, 2016). This shows that big data analytics capabilities alone cannot be expected to increase business model innovation and technological orientation is also required.

In China, Zhou et al. (2005) have found that the stronger an enterprise’s technological orientation the larger the advantage offered by its innovation. Similar research has revealed that businesses that are more technology-oriented generate product innovations that are more radical (Gatignon and Xuereb, 1997). This is because technological orientation has a strong impact on how effectively an employee uses information (Hunter and Perreault Jr, 2006; Sundaram et al., 2007). So the information extracted through big data should be expected to improve business model innovation more when there is mediation by a technological orientation.

Relevant studies have investigated mediating variables in the relationship between big data capabilities and innovation, which shows the importance of such factors. Mikalef et al. (2019) have found that big data capabilities can improve dynamic capabilities which have a positive impact on innovation. So dynamic capabilities are a mediator in the relationship between big data capabilities and innovation. They have also found that when there is high environmental diversity, the impact of big data capabilities on innovation is stronger. Su et al. (2021) have found that big data analytics capabilities positively affect performance of Chinese organizations. They have also found that dual innovations mediate this relationship, strengthening the effect of capabilities on outcomes. Related research has showed that technology adoption helps businesses achieve innovation (Cuevas-Vargas et al., 2022).

Based on these findings, our next hypothesis is

*H4*: Technological orientation mediates the relationship between big data analytics capabilities and business model innovation

### The direct and moderating effects of employee creativity

Creativity is valuable in the context of innovation (Joas and Beckert, 2002; El-Kassar et al., 2022). Creativity can be defined as the production of novel and useful ideas (Amabile, 1988). Creative ideas are the starting point of innovations (Amabile et al., 1996). Employee creativity positively affects an organization’s innovation capability because creative employees provide ideas that are raw materials for organizational innovation (Oldham and Cummings, 1996; Zhang and Bartol, 2010). This shows that employee creativity can have a direct positive effect on business model innovation. Since managers are also employees of a business, their creativity can also be expected to increase innovation. This is why Martensen and Dahlgaard (1999) have suggested organizations build cultures that encourage and effectively manage creativity. Supporting research has revealed that organisational outcomes are impacted by managerial characteristics (Hambrick and Mason, 1984).

Research has provided useful findings on this topic. Saether (2019) has found that when an organization supports creativity amongst employees, there is an increase in innovation. Chaubey et al. (2019) have found a positive relationship between employee creativity and organizational innovation. Creative employees supply a business with valued inputs for development and utilization of new processes, products and services (Bharadwaj and Menon, 2000). It has been observed that creative employees are likely to become role models for other employees, which leads to the latter also becoming idea generators (Shafique et al., 2019). This is an important finding because novel ideas often need to be mixed, for a breakthrough (Tushman and Anderson, 1986). So if there is a community of creative employees in an enterprise, there is a high probability of innovation (Tushman and Anderson, 1986). In China also, employee creativity has been found to be positively related to business innovation (Jiang et al., 2012).

Interestingly, exposure to foreign cultures improves creative performance of employees (Leung and Chiu, 2010; Maddux et al., 2010) and multicultural experiences increase creativity (Leung and Chiu, 2010). This type of creativity has been found to be positively related to business innovation in China (Jiang et al., 2012). This means that if employees possess foreign education or work experience, they are more likely to achieve business model innovation.

The main advantage of data-driven business model innovation is found in the opportunity to rationalize management’s intuitions and creativity (Cheah and Wang, 2017). Cao et al. (2019) have stated that creativity is necessary for leveraging big data because businesses must develop new analysis methods, which lead to understanding and implementation of new procedures. Therefore, it can be stated that employee creativity plays a moderating role in the relationship between big data analytics capabilities and business model innovation.

Based on this discussion, our next hypotheses are

*H5*: Employee creativity has a direct and positive effect on business model innovation

*H5a*: Employee creativity moderates the relationship between big data analytics capabilities and business model innovation so that this link is stronger at higher creativity levels

### Control variables

Research has found that businesses with different sizes adopt dissimilar innovation strategies (Damanpour, 1992) so business size has been included in our model as a control variable. One possible explanation is that larger enterprises have more resources for undertaking research and development (Clark, 2004; Fey and Birkinshaw, 2005). Past research has also discovered that adoption of innovation varies across industries (Bianchi et al., 2011). A possible reason is that organizations in different industries have divergent cultural values that influence their innovation efforts. So the industry of each business has been included as a control factor. Internationalization of enterprises has been found to impact performance, as well (Jantunen et al., 2008). One explanation could be that businesses fine-tune their strategies as the internationalization process happens and knowledge is gained (Jones and Coviello, 2005; Spence and Crick, 2009). Research has also found that employees’ foreign experience is positively related to business innovation (Yuan and Wen, 2018). Both foreign education and work experience positively impact innovation (Yuan and Wen, 2018). This is why firm internationalization is a relevant control variable for our model. Important differences in the elements of Business-to-Business (B2B) and Business-to-Consumer (B2C) markets lead to differences in innovation (Dotzel and Shankar, 2019) which shows that a business’s customer type can impact its performance in terms of innovation. So the customer type is a relevant control factor for our research. Relevant past research has also incorporated these control variables (Crespo Marquez et al., 2020).

## Materials and methods

### Pilot survey and instrumental design

The first set of questionnaires was in both Chinese and English. Before administering the final version, we conducted a pilot survey. For this purpose, questionnaires were administered to 30 Chinese and 25 foreign managers. Based on the feedback obtained through this and observations of Chinese language patterns, the final questionnaire was developed. It was administered online and included two parts. The first covered our model’s independent, dependent, mediating and moderating variables while the second covered our control variables. For this research, data was collected in the period from January to July 2021.

### Sampling technique

The minimum sample size is 212 for conducting SEM^1^ so our sample size is appropriate. Based on the findings of Hair et al. (2017b) and Cohen’s power theory (Cohen, 2013), we have assessed sample size adequacy. To confirm this sample’s statistical strength, we used the G*power post-hoc test for exogenous factors (with a significance level of 0.05), an effect size of 0.15 and a sample size of 380 (Statista, 2020). There were 4,154 companies listed on the Chinese stock exchange in 2020.^2^ We used the random sampling method to select 700 from these, representing the population of active local and multinational companies registered in China. This sample comprised mid-level and senior Chinese managers working at multinational companies (40%) and foreign managers at local companies (60%). We distributed 700 questionnaires (1 per organization) out of which 499 were completely filled. Only the complete ones were used for data analysis, so the response rate was 71.29%.

### Measures

For measuring the constructs in our model, we utilized instruments used in relevant studies (Table 1). Big Data Analytics Capabilities was measured using 13 items, out of 15 (Ravichandran et al., 2005; Aydiner et al., 2019a, 2019b). Two items were dropped because their factor loadings were below 0.5 (Hair et al., 2010). For this construct, the value of Cronbach’s Alpha was 0.958. Business Model Innovation was measured with 5 statements (Asemokha et al., 2019) and Cronbach’s Alpha was 0.911. Technological Orientation was measured using 4 items (Gatignon and Xuereb, 1997) and Cronbach’s Alpha was 0.826. Employee Creativity was measured with 9 statements (Ettlie and O'Keefe, 1982; Tierney et al., 1999). For this construct, the value of Cronbach’s Alpha was 0.942. For these four constructs, a 5-point Likert scale from 1 (strongly disagree) to 5 (strongly agree) was used. Business size was represented by the number of employees (Naqshbandi, 2018). For the industry type, respondents had to choose the sector in which their business was operating (Naqshbandi, 2018). The degree of internationalization was represented by Total Foreign Revenue divided by Total Assets, in terms of US$ (Riahi-Belkaoui, 1999). For the customer type, respondents were asked whether their organizations served businesses or final consumers (Crespo et al., 2020).

**Table 1:** Summary statistics.

Variables	Number (N)	Percentage (%)
Age		
18–30	50	10.02%
31–45	242	48.49%
46–60	120	24.05%
>60	87	17.43%
Gender		
Male	270	54.11%
Female	229	45.89%
Industry experience		
Less than 1 year	12	2.40%
1–5 years	81	16.23%
6–10 years	116	23.25%
Greater than 10 years	290	58.11%
Industrial Sector		
Manufacturing	142	28.46%
Services	176	35.27%
Trade	56	11.22%
Bank and Financial Institutions	48	9.62%
E-commerce	77	15.43%
Company positions		
Chief Executive Officer	40	8.02%
Business Unit Head	53	10.62%
Senior Manager	189	37.87%
Functional Manager	121	24.25%
Other top management positions	96	19.24%
Size of the firm		
1–50 employees	143	28.66%
51–150 employees	112	22.44%
151–250 employees	87	17.43%
251–500 employees	72	14.42%
Greater than 500 employees	85	17.03%
Degree of firm internationalization		
0–4.99 million USD	111	22.24%
5–9.99 million USD	127	25.45%
10–14.99 million USD	107	21.44%
15–19.99 million USD	94	18.83%
Greater than 20 million USD	60	12.02%
Types of customers served		
Business to Business	275	55.11%
Business to Customer	224	44.89%

### Common method and non-response bias

Participation in this study was voluntary and the confidentiality of responses was guaranteed (Podsakoff, 2003), to lower the risk of Common Method Bias (CMB). Harman’s one-factor test was conducted to detect CMB (Harman, 1976). We found that the first factor accounted for 47.30% of the variance which is lower than the 50% limit so there is no evidence of CMB (Fuller et al., 2016). To address concerns about CMB, we also utilized established scales and divided the questionnaire into 5 parts, 4 covering our model variables and 1 covering the control variables (Ciampi et al., 2021).

We also compared the size and age of participating and non-participating businesses in our sample. ANOVA provided *p* values equal to 0.892 and 0.651 respectively, showing that there was no statistically significant difference. We also conducted t-tests, comparing the age and size of early and late responding firms (Armstrong and Overton, 1977). We did not find any statistically significant difference (p values 0.512 and 0.392, respectively).

## Results

### Data analysis technique

SEM is a method that is appropriate for analyzing data to identify relationships between variables (Purwanto et al., 2021). In recent years the number of published articles using Partial Least Squares SEM (PLS-SEM) has increased significantly, compared with Covariance-Based SEM (CB-SEM; Hair et al., 2017b). PLS-SEM is now applied in many social science areas, including organizational management (Sosik et al., 2009). An important reason for this trend is that when using PLS-SEM researchers benefit from the higher statistical power, compared with CB-SEM (Reinartz et al., 2009; Hair et al., 2017b). This means that PLS-SEM is more likely to highlight relationships as significant when they are indeed present in the population (Sarstedt and Mooi, 2019). We selected PLS-SEM for data analysis after taking these factors into account. We applied PLS-SEM in the software SmartPLS v3 (Hair et al., 2014; Sarstedt et al., 2019). For hypothesis testing, bootstrapping was used. Moreover, we have used 5,000 bootstrap samples and the two-tailed test.

### Descriptive statistics

Results of our study are based on a sample of 499 respondents whose characteristics are presented in Table 1. The sample composition was 54.11% males and 45.89% females. Most of the respondents (48.49%) were in the age group from 31 to 45 years. Amongst the participants, 58.11% had more than 10 years of experience in their industry. Most belonged to the service (35.27%) and manufacturing (28.46%) sectors. In the context of organizational positions, 37.87% of respondents were senior managers and 24.25% were functional managers. In the context of size, 28.66% of the businesses had 1–50 employees and 17.03% had more than 500 employees. More than a quarter of the sample (25.45%) had firm internationalization of 5–9.99 million USD. Business customers were served by 55.11% of the sample while final consumers were served by 44.89%.

### Reliability and validity test

It is important to check the reliability and validity of measurement tools utilized in a study. Construct reliability and composite reliability have been assessed (Brown, 2002). The values are provided in Table 2. All are higher than the suggested threshold of 0.70 (Nunally and Bernstein, 1978). The Average Variance Extracted (AVE) values, also given in Table 2, have been used to assess convergent validity. The values are acceptable when compared with the generally used threshold of 0.50 (Henseler et al., 2016). Multicollinearity has been checked (Aiken et al., 1991) and shown in Table 2 in the form of Variance Inflation Factor (VIF) values. The values for all constructs are less than 5 and, therefore, acceptable (Ringle et al., 2015). Values of Rho_A are between Cronbach’s Alpha and composite reliability so are acceptable.

**Table 2:** Factor loadings, reliability, validity and multicollinearity.

Constructs	Loadings	Cronbach’s alpha (CA)	Rho_A	Composite reliability (CR)	Average variance extracted (AVE)	Variance inflation factor (VIF)
Big data analytical capabilities (BDA)		0.958	0.961	0.963	0.667	1.099
BDA 1	0.854					
BDA 2	0.819					
BDA 3	0.798					
BDA 4	0.771					
BDA 5	0.833					
BDA 6	0.827					
BDA 7	0.839					
BDA 8	0.823					
BDA 9	0.846					
BDA 10	0.774					
BDA 11	0.826					
BDA 12	0.804					
BDA 13	0.797					
Technological orientation (TCO)		0.826	0.834	0.884	0.657	1.089
TCO 1	0.859					
TCO 2	0.803					
TCO 3	0.766					
TCO 4	0.811					
Employee creativity (EMC)		0.942	0.944	0.951	0.682	1.025
EMC 1	0.858					
EMC 2	0.834					
EMC 3	0.849					
EMC 4	0.822					
EMC 5	0.818					
EMC 6	0.838					
EMC 7	0.806					
EMC 8	0.821					
EMC 9	0.785					
Business model innovation (BDI)		0.911	0.914	0.934	0.738	
BDI 1	0.858					0.760
BDI 2	0.866					0.817
BDI 3	0.856					0.846
BDI 4	0.846					0.821
BDI 5	0.870					0.756

Discriminant validity has been assessed using the Fornell Larcker criterion and the Heterotrait Monotrait (HTMT) ratio, shown in Tables 3, 4 respectively. Both criteria are commonly recognized and other researchers have applied them (Henseler et al., 2016; Neneh, 2019a). Discriminant validity is defined as the square root of AVE (Fornell and Larcker, 1981) and HTMT values must be less than 0.85 (Henseler et al., 2016). The highest HTMT value is 0.123 which shows that all constructs possess discriminant validity.

**Table 3:** Discriminant validity (Fornell Larcker).

	BDA	BDI	EMC	TCO
BDA	**0.817**	-	-	-
BDI	0.275	**0.859**	-	-
EMC	0.141	0.263	**0.826**	-
TCO	0.278	0.261	0.104	**0.810**

Values in diagonals are square roots of AVE. Values under diagonals are correlations. BDA = big data analytic capabilities, TCO = technological orientation, EMC = employee creativity, and BDI = business model innovation.

**Table 4:** Discriminant validity (HTMT).

	BDA	BDI	EMC	TCO
BDA	-	-	-	-
BDI	0.289	-	-	-
EMC	0.148	0.281	-	-
TCO	0.305	0.296	0.123	-

BDA = big data analytic capabilities, TCO = technological orientation, EMC = employee creativity, and BDI = business model innovation.

### Structural model

**Figure 1:**
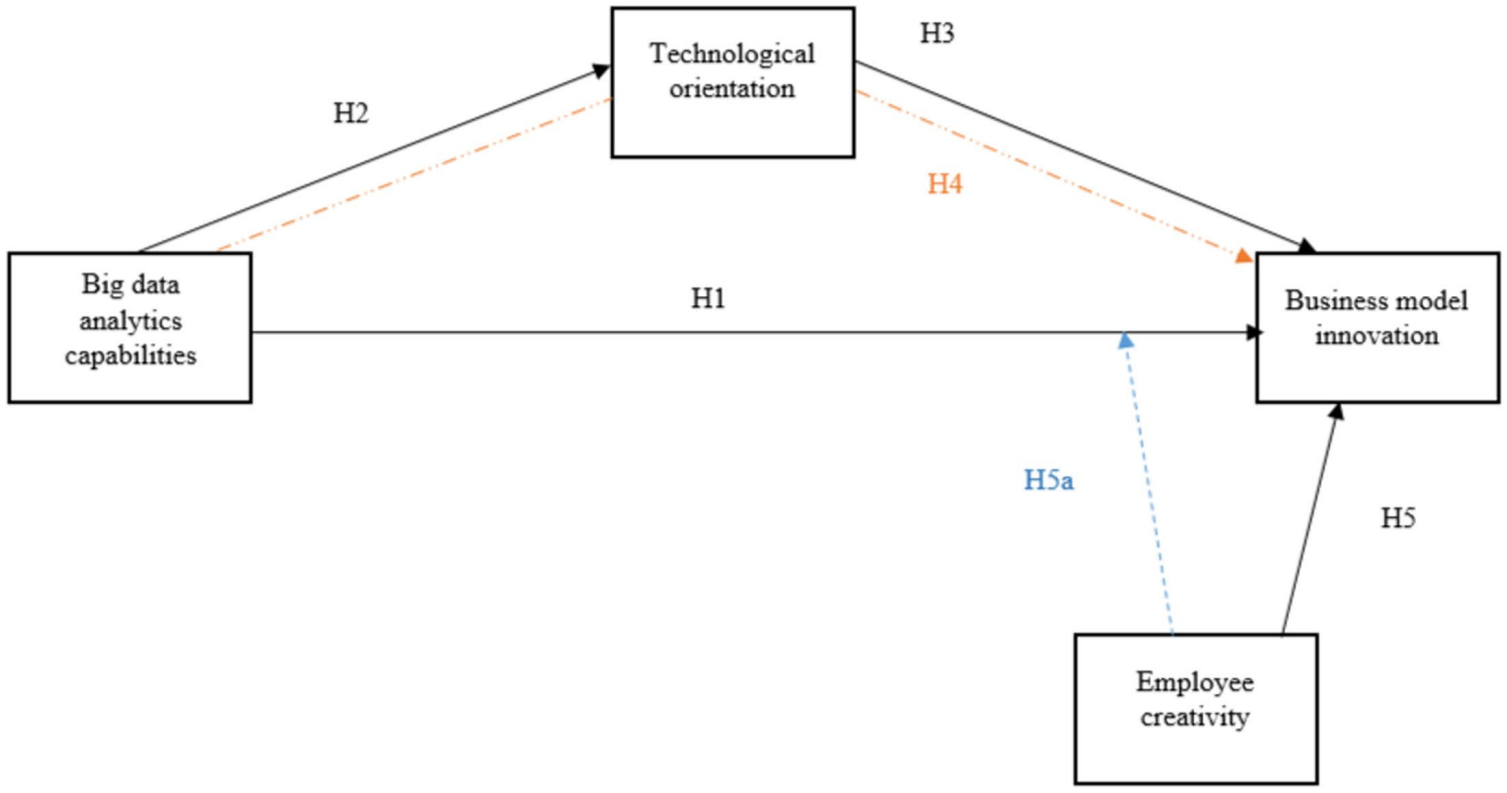
Conceptual model.

SmartPLS v3 and the PLS algorithm approach have been deployed to analyze the structural model. The standardized root mean square residual value has been used to assess model fit, with a suggested value of 0.08 (Henseler et al., 2016). This model’s value is 0.048, indicating the model’s overall fitness. Figure 1 presents the R^2^ value, which shows that this model explains 15.90% of the variance in business model innovation. This R^2^ value is acceptable because it is not below 10% (Falk and Miller, 1992).

**Figure 2:**
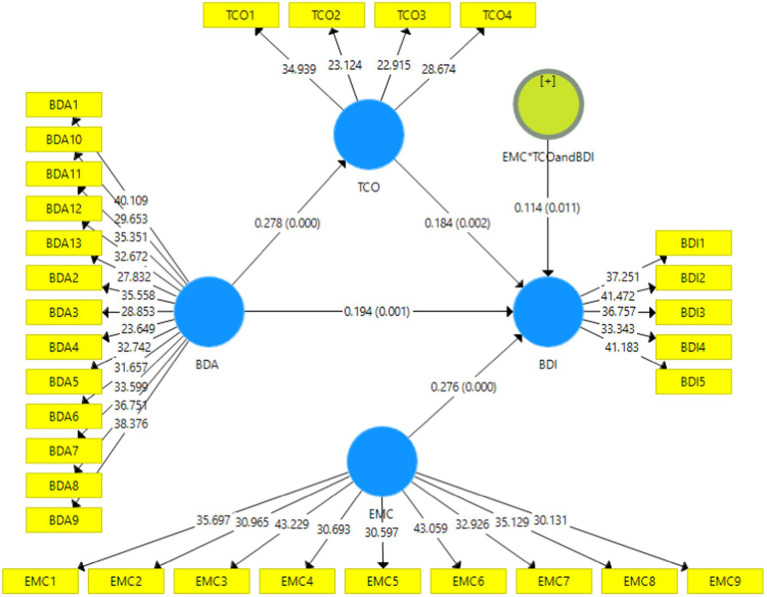
Structural model. BDA = big data analytic capabilities, TCO = technological orientation, EMC = employee creativity, and BDI = business model innovation.

**Figure 3:**
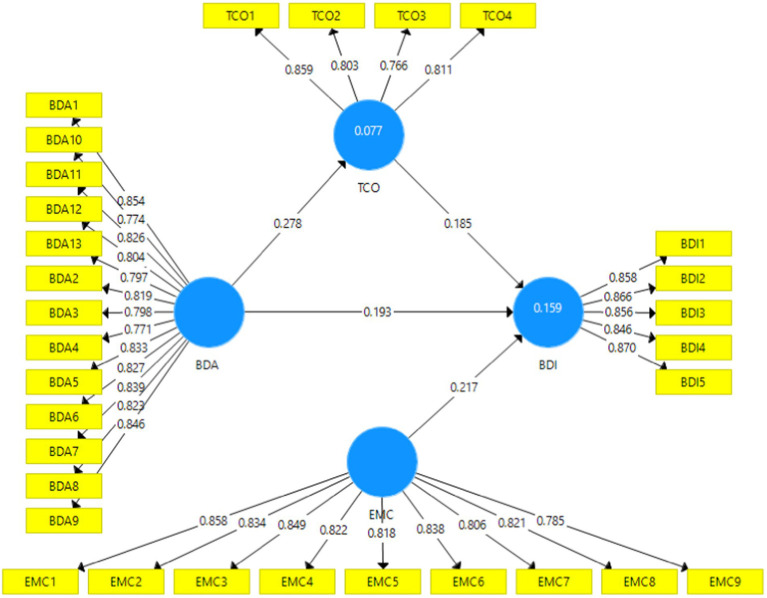
Measurement model. BDA = big data analytic capabilities, TCO = technological orientation, EMC = employee creativity, and BDI = business model innovation.

All the direct-effect hypotheses have been accepted. These results are shown in Table 5. Big data analytics capabilities have a significant and positive impact on business model innovation (*β* = 0.194**, *t* = 3.252, *p* < 0.01), providing support for H1. Big data analytics capabilities also have a significant positive effect on technological orientation (*β* = 0.278**, *t* = 5.033, *p* < 0.01) so H2 is accepted. There is significant and positive impact of technological orientation on business model innovation (*β* = 0.184*, *t* = 3.189, *p* < 0.01) so H3 is supported. There is a significant positive effect of employee creativity on business model innovation (*β* = 0.276**, *t* = 4.491, *p* < 0.01) so H5 has been accepted (Figures 2, 3).

**Table 5:** Structural model estimates.

Hypotheses	Relationships	Standardized paths (*β*)	*t*-statistics	*p*-values	Hypotheses accepted/not accepted
H1	BDA -> BDI	0.194**	3.252	0.001	Accepted
H2	BDA -> TCO	0.278**	5.033	0.000	Accepted
H3	TCO -> BDI	0.184*	3.189	0.002	Accepted
H5	EMC -> BDI	0.276**	4.491	0.000	Accepted

BDA = big data analytic capabilities, TCO = technological orientation, EMC = employee creativity, and BDI = business model innovation.

** *p* < 0.01;

* *p* < 0.05.

### Measurement model

#### Mediating effect

Technology orientation positively partially mediates the relationship between big data analytics capabilities and business model innovation (*β* = 0.051*, *t* = 2.450, *p* < 0.05), providing support for H4. The result is provided in Table 6, with Percentile Confidence Interval (PCI) and Variance Accounted For (VAF) values. To test for mediation 5,000 bootstrap sample were used along with two-tailed testing. To determine the strength of mediation effect Variance Accounted For (VAF) is computed. VAF value shows the ratio of beta co-efficient of indirect effect to the total effect. In SmartPLS software VAF is not calculated automatically therefore we have used the manual calculation to compute it by extracting the values from PLS algorithm. Moreover, the detail calculation is provided in Table 6 notes. If the VAF values comes out to be greater than 80% then it indicates a full mediation, if value lies between 20 and 80% it indicates partial mediation and if the value falls below 20% it indicates no mediation (Hair et al., 2011). The VAF value for this study is 20.8% which lies between 20 and 80% interval so there is partial mediation.

**Table 6:** Indirect Effects (mediation effects).

Hypotheses	Relationship	Standardized paths (*β*)	T-statistics	P-values	2.5%	97.5%	VAF	Hypotheses accepted/not accepted
H4	BDA -> TCO -> BDI	0.051*	2.450	0.015	0.015	0.099	0.208	Accepted

BDA = big data analytic capabilities, TCO = technological orientation, EMC = employee creativity, and BDI = business model innovation. VAF = [(axb)/(axb + c)] = [(0.27×0.184)(0.27×0.184 + 0.194)] where a = BDA-TCO, b = TCO-BDI and c = BDA-BDI.

#### Moderating effects

**Figure 4:**
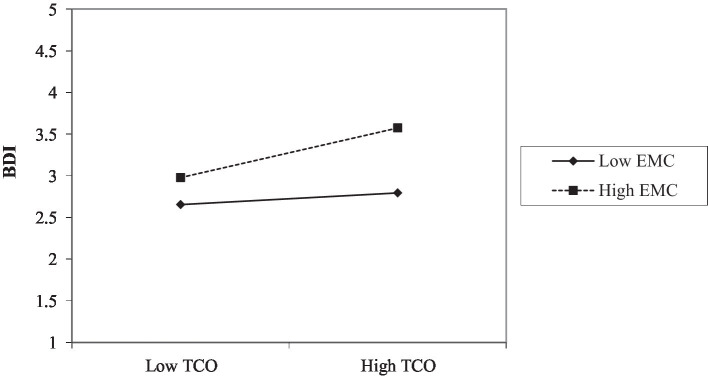
Moderating effect (EMC*TCO and BDI).

Employee creativity moderates the relationship between big data analytics capabilities and business model innovation (*β* = 0.114, *t* = 2.563, *p* < 0.05), providing support for H5a. Figure 4 further shows the moderating effect (Table 7).

**Table 7:** Moderating effects.

Hypotheses	Relationships	Standardized paths (*β*)	*t*-statistics	*p*-values	Hypotheses accepted/not accepted
H5a	EMC*TCO and BDI -> BDI	0.114*	2.563	0.011	Accepted

BDA = big data analytic capabilities, TCO = technological orientation, EMC = employee creativity, and BDI = business model innovation.

To obtain meaningful and reliable results, we have also evaluated the model’s predictive power using Stone-Geisser’s Q^2^. It is a cross validity redundancy check performed using a blindfolding procedure. It particularly set omission distance of 7 as a criterion for predictive relevance (Janadari et al., 2016). The general rule is that Q^2^ values above 0 show the respective predictive relevance (Janadari et al., 2016). We have acquired the Q^2^ value by using the structural equation model. The Q^2^ value, presented in Table 8, is 0.126 which shows predictive relevance (Janadari et al., 2016).

**Table 8:** Q^2^ for model predictive relevance.

	SSO	SSE	Q^2^ (=1-SSE/SSO)
BDA	4511.000	4511.000	
BDI	1735.000	1515.850	0.126
EMC	3123.000	3123.000	
EMC*TCO and BDI	347.000	347.000	
TCO	1388.000	1324.683	0.046

## Discussion

There is a lack of empirical research on how big data can be utilized to achieve business innovation (Ciampi et al., 2021). Therefore, our empirical study has made a valuable contribution to existing knowledge about the impact of big data capabilities on business model innovation. It has also provided information about the roles of technological orientation and employee creativity, in this context. Our research has revealed that big data analytics capabilities increase business model innovation, which is consistent with results obtained before the pandemic (Kiron et al., 2012, 2014; Shamim et al., 2020). Big data capabilities strengthen technological orientation, aligned with results of studies before the pandemic (Mandal, 2018; Wang et al., 2021). Technological orientation increases innovation. This finding is also aligned with research completed before COVID-19 (Ravichandran et al., 2017; Saldanha et al., 2017; Dong and Yang, 2019). Technology orientation mediates the relationship between big data capabilities and innovation. This is supported by the work of Limbu et al. (2014), Sundaram et al. (2007) and Zhou et al. (2005) completed before COVID-19. We have found that employee creativity boosts business innovation. It also strengthens the positive relationships between big data capabilities and innovation. These findings are supported by studies conducted before the COVID-19 pandemic (Chaubey et al., 2019; Saether, 2019).

### Theoretical implications

This study has made multiple conceptual contributions. First, it has added to existing literature on the benefits of dynamic capabilities for businesses (Akter et al., 2016; Mikalef et al., 2019). Second, we have responded to calls for more research on the internal drivers of business model innovation (Martins et al., 2015; Saebi and Foss, 2015; Foss and Saebi, 2017; Frankenberger and Sauer, 2019). These drivers include technological orientation and employee creativity (Ciampi et al., 2021). Further, to the best of our knowledge, no past study has included both mediating and moderating variables in the relationship between big data analytics capabilities and business model innovation. Third, the study findings have provided support for the dynamic capabilities view. To the best of our knowledge, past studies have not analyzed this conceptual model in the Chinese context. Therefore, this research has made a contextual contribution as well.

### Practical implications

Our findings show that to achieve business model innovation, managers should utilize big data and related technologies. They should also hire and train employees who are most capable of utilizing such resources. Business management should adopt and promote a technological orientation, in this context. Another important implication is that organizations should try to select and support employees who show creativity (Ciampi et al., 2021). Further, such workers should be placed in management positions as they are likely to make positive contributions to business model innovation. In this setting, top managers can play an important role in decisions such as those related to technology infrastructure and recruitment of competent data professionals (Barton and Court, 2012). During the ongoing COVID-19 pandemic, business model innovation is even more important because it is a recovery option for enterprises that have been affected negatively.

Our research has certain limitations which should be viewed as opportunities for future studies. First, a survey with self-reporting was used which means that the threat of cognitive bias exists so future studies should use different methodologies. Second, our work was conducted in China only so researchers should analyze these relationships in different settings to find out if the associations hold. Multigroup analysis can be conducted, based on factors such as industrial sectors and developed/developing economies. This comparative analysis will add to existing knowledge. Third, other mediating variables can be investigated, such as learning orientation, in the relationship between big data capabilities and business performance (Ciampi et al., 2021). Competitive advantage (Anwar et al., 2018) and big data value creation (Shamim et al., 2020) can also play mediating roles. Important moderating factors include resistance to change (Shahbaz et al., 2019), risk tolerance (Hock-Doepgen et al., 2021) and the organizational environment (Mikalef et al., 2019) so these should be investigated to better understand the association between big data capabilities and business performance. Fourth, future researchers can assess the innovation performance of government and humanitarian organizations for a unique view of this topic.

## Conclusion

Organizations that use big data analytics can achieve higher levels of performance, including business model innovation. The purpose of this research was to analyze the relationships between big data analytics capabilities and education, business model innovation, technological orientation and employee creativity. Online questionnaire data from a sample of 499 managers at Chinese enterprises was analyzed though PLS-SEM. It has been found that big data analytics capabilities strengthen technological orientation and increase business model innovation. Technological orientation also improves innovation. Employee creativity strengthens the positive relationship between big data capabilities and innovation. These results have provided multiple implications and created opportunities for future researchers. Our findings show that to achieve business model innovation, managers should utilise big data and other technologies. They should hire and train employees who are most capable of utilising such resources. Moreover, organizations should collaborate with education sector especially universities to foster big data analytics basic knowledge at the undergraduate and post graduate curriculum. Business management should adopt and promote a technological orientation. Organizations should try to select and support employees who show creativity (Ciampi et al., 2021). During the ongoing COVID-19 pandemic, innovation is even more important because it is a recovery option for businesses that have been affected negatively.

## Data availability statement

The raw data supporting the conclusions of this article will be made available by the authors, without undue reservation.

## Ethics statement

The Jiangsu University review board exempted the research from ethical approval as it was a survey-based study. Informed consent was obtained from all subjects involved in the study while collecting the data through an online questionnaire.

## Author contributions

YC and SF contributed to developing the theoretical framework, data analysis, and overall writing of the manuscript. AA, MA, and ZA contributed to data collection and the writing, editing, and organization of the manuscript. All authors contributed to the article and approved the submitted version.

## Funding

This work was supported by General Program of the Chinese Society of Academic Degrees and Graduate Education for Research and Practice of the Promotion of the Quality of Overseas Engineering Postgraduate Students Education through the Integration between Industry and University under Fund Serial Number (2020MSA350), China Education Association for International Exchange for Research and Practice of the Promotion of the Quality of Overseas Engineering Postgraduate Students Education through the Integration among Government, Industry, and University under Fund Serial Number (Jixieyan 2021-008), Chinese Society of Educational Development Strategy for Research of the Promotion of the Global Competence Cultivation of the Students from University relevant to agriculture through the Integration among Government, Industry and University under Fund Serial Number (SRB202131), and Major Program of the Chinese Society of Academic Degrees and Graduate Education for Exploration and Practice of the Cultivation Mode of the Students from Belt and Road Countries under Fund Serial Number (2020ZAC11).

## Conflict of interest

The authors declare that the research was conducted in the absence of any commercial or financial relationships that could be construed as a potential conflict of interest.

## Publisher’s note

All claims expressed in this article are solely those of the authors and do not necessarily represent those of their affiliated organizations, or those of the publisher, the editors and the reviewers. Any product that may be evaluated in this article, or claim that may be made by its manufacturer, is not guaranteed or endorsed by the publisher.

## Footnotes

^1^ https://www.danielsoper.com/statcalc/calculator.aspx?id=89

^2^ https://www.statista.com/statistics/225725/number-of-companies-listed-on-the-chinese-stock-exchange/
