# Supplementary material for: The influence of big data analytic capabilities building and education on business model innovation
Source: Front Psychol. 2022 Oct 18;13:999944. doi: 10.3389/fpsyg.2022.999944 (PMC9623004; doi:10.3389/fpsyg.2022.999944)
Supplement: Supplementary file 1 [file Table_1.DOCX]

**Appendix 1:** Constructs and items used in this study

| **Constructs** | **Items** | **References** |
| --- | --- | --- |
| Big data analytics capabilities | Our IS infrastructure is strong enough between inter-organisational units | Aydiner et al., 2019a, 2019b; Ravichandran and Lertwongsatien, 2005 |
|  | Our IS infrastructure is suitable for developing customised software applications when the need  Arises |  |
|  | Our IS infrastructure can respond quickly to requests from internal and external customers |  |
|  | Our IS infrastructure capacity can handle multiple applications |  |
|  | Our IS infrastructure provides fast and flexible operations for the internet-based system |  |
|  | Our IS staff has adequate knowledge of the computer-based system |  |
|  | Our firm seeks a high degree of computer-based technical expertise for the IS department's employees |  |
|  | Our IS staff can implement the right application at the right time |  |
|  | Our IS staff can discover potential problems rapidly in the system |  |
|  | Our IS staff is capable of quickly maintaining the system whenever a failure occurs |  |
|  | Our firm's IS strategy is in line with our corporate strategy |  |
|  | Our firm's IS managers have an executive-level authority |  |
|  | Our IS processes are well defined and documented |  |
|  | Our IS department has a clear guideline on how to prioritise service requests from users |  |
|  | Our IS department has a clear guideline on how to use IT resources in our firm |  |
| Business model innovation | When necessary, we are able to carry out massive internal reconfigurations to enhance our overall value proposition to our customers | Asemokha et al. (2019) |
|  | When we sense an opportunity, we are quick at re-organising our operating processes |  |
|  | When necessary, we are able to re-organise our partner network to improve our value proposition to our customers |  |
|  | New opportunities to serve our customers are quickly understood |  |
|  | We regularly consider innovative opportunities for changing our existing pricing models |  |
| Technological orientation | We use advanced technologies in our product development | Gatignon and Xuereb (1997) |
|  | Our products always contain the latest technology |  |
|  | We are actively developing new technologically advanced products |  |
|  | Technological innovation based on research is accepted without further ado in our company |  |
| Employee creativity | Demonstrated originality in his/her work | Ettlie and O’Keefe (1982)  Tierney et al. (1999) |
|  | Took risks in terms of producing new ideas in doing the job |  |
|  | Found new uses for existing methods or equipment |  |
|  | Solved problems that had caused others difficulty |  |
|  | Tried out new ideas and approaches to problems |  |
|  | Identified opportunities for new products/processes |  |
|  | Generated novel but operable work-related ideas |  |
|  | Served as a good role model for creativity |  |
|  | Generated ideas revolutionary to our field |  |

**Appendix 2: Questionnaire**

**This questionnaire is a part of the requirements for research. Please fill this in to the best of your knowledge. The information collected will be kept confidential. Thank you for your help in our research endeavor.**

Please indicate your level of agreement with each statement.

|  |  | 1 | 2 | 3 | 4 | 5 |
| --- | --- | --- | --- | --- | --- | --- |
|  |  | Strongly disagree | Disagree | Neither disagree nor agree | Agree | Strongly agree |
|  | Infrastructure capabilities |  |  |  |  |  |
| BDA1 | Our IS infrastructure is strong enough between inter-organisational units |  |  |  |  |  |
| BDA2 | Our IS infrastructure is suitable for developing customised software applications when the need  Arises |  |  |  |  |  |
| BDA3 | Our IS infrastructure can respond quickly to requests from internal and external customers |  |  |  |  |  |
| BDA4 | Our IS infrastructure capacity can handle multiple applications |  |  |  |  |  |
| BDA5 | Our IS infrastructure provides fast and flexible operations for the internet-based system |  |  |  |  |  |
|  | Human resource capabilities |  |  |  |  |  |
| BDA6 | Our IS staff has adequate knowledge of the computer-based system |  |  |  |  |  |
| BDA7 | Our firm seeks a high degree of computer-based technical expertise for the IS department's employees |  |  |  |  |  |
| BDA8 | Our IS staff can implement the right application at the right time |  |  |  |  |  |
| BDA9 | Our IS staff can discover potential problems rapidly in the system |  |  |  |  |  |
| BDA10 | Our IS staff is capable of quickly maintaining the system whenever a failure occurs |  |  |  |  |  |
|  | Management capabilities |  |  |  |  |  |
| BDA11 | Our firm's IS strategy is in line with our corporate strategy |  |  |  |  |  |
| BDA12 | Our firm's IS managers have an executive-level authority |  |  |  |  |  |
| BDA13 | Our IS processes are well defined and documented |  |  |  |  |  |
| BDA14 | Our IS department has a clear guideline on how to prioritise service requests from users |  |  |  |  |  |
| BDA15 | Our IS department has a clear guideline on how to use IT resources in our firm |  |  |  |  |  |
|  |  |  |  |  |  |  |

Please indicate your level of agreement with each statement.

|  |  | 1 | 2 | 3 | 4 | 5 |
| --- | --- | --- | --- | --- | --- | --- |
|  |  | Strongly disagree | Disagree | Neither disagree nor agree | Agree | Strongly agree |
| BDI1 | When necessary, we are able to carry out massive internal reconfigurations to enhance our overall value proposition to our customers |  |  |  |  |  |
| BDI2 | When we sense an opportunity, we are quick at re-organising our operating processes |  |  |  |  |  |
| BDI3 | When necessary, we are able to re-organise our partner network to improve our value proposition to our customers |  |  |  |  |  |
| BDI4 | New opportunities to serve our customers are quickly understood |  |  |  |  |  |
| BDI5 | We regularly consider innovative opportunities for changing our existing pricing models |  |  |  |  |  |

Please indicate your level of agreement with each statement.

|  |  | 1 | 2 | 3 | 4 | 5 |
| --- | --- | --- | --- | --- | --- | --- |
|  |  | Strongly disagree | Disagree | Neither disagree nor agree | Agree | Strongly agree |
| TCO1 | We use advanced technologies in our product development |  |  |  |  |  |
| TCO2 | Our products always contain the latest technology |  |  |  |  |  |
| TCO3 | We are actively developing new technologically advanced products |  |  |  |  |  |
| TCO4 | Technological innovation based on research is accepted without further ado in our company |  |  |  |  |  |

Please indicate how often the following statements characterise you.

|  |  | 1 | 2 | 3 | 4 | 5 |
| --- | --- | --- | --- | --- | --- | --- |
|  |  | Strongly disagree | Disagree | Neither disagree nor agree | Agree | Strongly agree |
| EMC1 | I have demonstrated originality in my work |  |  |  |  |  |
| EMC2 | I have taken risks in terms of producing new ideas in doing the job |  |  |  |  |  |
| EMC3 | I have found new uses for existing methods or equipment |  |  |  |  |  |
| EMC4 | I have solved problems that had caused others difficulty |  |  |  |  |  |
| EMC5 | I have tried out new ideas and approaches to problems |  |  |  |  |  |
| EMC6 | I have identified opportunities for new products/processes |  |  |  |  |  |
| EMC7 | I have generated novel but operable work-related ideas |  |  |  |  |  |
| EMC8 | I have served as a good role model for creativity |  |  |  |  |  |
| EMC9 | I have generated ideas revolutionary to our field |  |  |  |  |  |

**What is the size (employee number) of your business organisation?**

1. 1-50 b) 51-150 c) 151-250 d) 251-500 e) More than 500

**Which industrial sector is the enterprise operating in?**

1. Manufacturing b) Services c) Trade d) Banks and financial institutions

e) E-commerce

**What is your average “degree of firm internationalisation” (Foreign revenue over Total Assets) in the last 5 years?**

1. 0-4.99 million USD b) 5-9.99 million USD c) 10-14.99 million USD

d)15-19.99 million USD e) More than 20 million USD

**What are the types of customers served by your business?**

1. Business to Business b) Business to Customer
